# On Medical Examinations

**Published:** 1829-01-01

**Authors:** 


### Medical Examinations.

Among the speculations which are daily vomited forth by the medical press respecting radical changes—declamations which are eminently calculated to prevent all moderate and rational reform, by deterring influential members of the profession from joining in the cry—we shall here notice the project of *testing* all candidates for medical diplomas or licences by the examination alone, without any production of documents respecting the time, place, or amount of previous medical education or study. A more wild, dangerous, and visionary plan was never engendered in the brain of the most fiery fanatic or levelling enthusiast. It argues a monstrous lack of knowledge of the world. In the first place, we challenge the proposers to shew any country in which it is acted on. Certainly not in those where the scrutiny is most rigorous—as, for example, in Paris. It is almost impossible that examinations can be carried on by tests more effectual for sifting the candidate's qualifications to the very bottom, than at Paris:—but does the Parisian Faculty dispense with the proofs of *how*, *when*, and *where* the knowledge was obtained? By no means. These documents are rigidly demanded, and must be produced before the ordeal is commenced. The Parisian examiners are men who have studied their profession, and who know the difference between learning and knowledge. They are well aware that the *mode* of acquiring information is a circumstance of the highest importance —and that the mere capability of answering questions, however *ingeniously* devised, does not constitute the criterion of knowledge. They have wisely, therefore, enforced the production of preliminary proofs, *how, when, and where* the knowledge was acquired. We shall now descend to a few particulars—appealing, not to the *chamber practitioner*, who issues his Utopian speculations from a heated imagination, but to those who, from actual observation and experience, are capable of judging on this point.

I. Pharmacy. No one will accuse us of advocating the present system of indenture, which we believe to be double, if not triple, the duration it ought to be. But we maintain that no man can practise either physic or surgery (much less pharmacy) well, who does not possess some practical knowledge, not merely of the properties, chemical constituents, and doses of medicinal agents, but of the *manipulation and extemporaneous composition* of the same, whether these last be learnt behind the counter of the chemist, in the private surgery of the general practitioner, or in the pharmacy of the hospital or dispensary. Now it is utterly impossible that a court of examiners can ask any pharmaceutical questions that may not be answered from what is taught in lectures on materia medica, or what is printed in the Dispensatories—unless, indeed, they keep a shop on one side of the Hall, and compel the candidates to boil decoctions, filter tinctures, roll pills, and compound mixtures. The certificate or proof of "actual service," in this department of the medical state, cannot, therefore, be dispensed with, unless we admit that pharmacy, learnt in books or lectures, is as good as that learnt by the actual practice of the same.

II. Anatomy. Of all the branches of medical or surgical science, anatomy is that which might be best ascertained by an examination. But we are convinced that, even here, the new or Utopian plan would break down. The whole of descriptive anatomy—the whole science of physiology, may be more *pertly* learnt from books, lectures, or even grinders, than by actual dissection. All *vivâ voce* questions, then, might be answered by means of the very worst species of study —*cramming*. But, says the reformer, we shall have skeletons, vascular and neurological subjects—dried muscular extremities on the table—and, by these, we shall find out the candidate's anatomical knowledge. Vain expectation! On all *these subjects*, the grinder can descant, and the student can get crammed. "Then (says the projector of the new scheme) we shall have *brains* (if such can be found) in every court of examiners, and each candidate shall demonstrate the various parts of the encephalon." Who shall find subjects—who shall find time for this species of manual anatomy? The thing is impracticable—and, if practicable, it ought not to supersede the proofs of the *quo modo* in which the anatomical knowledge is obtained.

III. SURGERY. The plan under consideration would tend to abridge the study of surgical diseases, by the toilsome and expensive mode of actual observation in hospitals and dispensaries; while it would encourage the accumulation of technical descriptions from books and "GRINDERS," in the former of which, the symptoms of surgical diseases, and the steps of surgical operations, are minutely laid down. This species of surgical erudition would make a better figure in the examination, than that which was acquired by ocular observation in hospitals, or by oral instructions from teachers. How much superior the *latter* is, we need not stop to prove.

IV. CLINICAL MEDICINE. This, the most important of all the medical studies, cannot be conveyed by means of books, or even of *vivâ voce* lectures—nor can any species of examination ascertain the candidate's proficiency in it—unless there was a ward of sick patients attached to the examination hall. The only security, then, for clinical study, is the written proof of hospital attendance, which proof the wild radical plan of reform would dispense with. This fact alone, which cannot be questioned, is sufficient to damn the proposed test of medical and surgical qualifications—a test which, if fully acted on, would do all in its power to depress the regular teacher—patronize the grinder—and imbue the medical student with technical, instead of practical, knowledge!

We have noticed this chimerical proposal, not from an idea that it will ever be entertained by any faculty, between Terra del Fuego and Lapland, but to shew the medical student the grounds of discontent, by which he is excited into hostility against those who are, by law, entrusted with his examination. That there is ample field for beneficial reform in our systems of medical education, in this country, we have often shewn; but the wild ravings of a few visionary reformers have driven the period for its investigation to an indefinite distance. This is already too plainly proved. No human power—no play on the human passions, could now congregate a dozen of *respectable* reformers in the Freemasons' Tavern—or draw half that number of petitions to Parliament from the united empire! Yet the same state of things now exists as in 1826. We are told, indeed, by the radical press, that peace and perfect unanimity obtain throughout all ranks of the profession; and that they only wait the return of Spring, to burst forth, in a storm of virtuous indignation, against their oppressors! This is a curious kind of tranquillity. The fact is, a few half-cracked agitators have made sensible men shrink from every kind of participation with them; and the agitators, being now left to themselves, they call it "PEACE!" "*Ubi solitudinem faciunt* PACEM *appellant.*" Under existing circumstances, there is not the remotest chance or hope of medical reform, except what may flow from the spontaneous concessions of the constituted authorities. Half a dozen medical Cobbetts have done more mischief than fifty medical Burdetts can repair! The corporate bodies now smile at their opponents, whose ranks are deserted, in consequence of leaders, with whom Falstaff's gang, with Bardolf at their head, would be ashamed to associate! Yet we think it would be wise in these corporate bodies, to seize this favourable opportunity for introducing liberal and enlightened measures, corresponding with the more extended views of modern times. It would be infinitely more graceful, that such measures should flow spontaneously from the constituted authorities, than that they should be exacted by popular clamour. A time may come, when the respectable members of the profession may think fit to form a strong and united phalanx, in favour of medical REFORM, after the ridicule and degradation attached to the word, and even to the measure, by the interference of the present advocates, shall have subsided. The voice of the profession must then be heard; and it must have a great moral force when divested of the "RIBALDRY" with which it is now associated.

We conclude with a suggestion to the Court of Examiners of the Apothecaries' Company, which, we are convinced, if acted on, would be of paramount importance. It is this:—let the proofs of lectures and hospital attendance be taken any time after the age of 17 years. The apprenticeship need not then be disturbed. Every father will take care to have an understanding with the apprentice's master, that the periods of study shall be *included* in the five years. This is all that is wanted at present.

---

### LII.

### Lumbar Abscess in a Horse.

